# A novel method for measuring sublingual temperature using conventional non-contact forehead thermometer

**DOI:** 10.12688/f1000research.74876.2

**Published:** 2022-02-18

**Authors:** Chakrapani Mahabala, Pradeepa H. Dakappa, Arjun R. Gupta

**Affiliations:** 1 Department of Internal Medicine, Kasturba Medical College,Mangalore, Manipal Academy of Higher Education, Manipal, Karnataka, 575001, India; 2 Department of Pharmacology, Nanjappa Hospitals, Durgigudi, Shivamogga, Karnataka, 577201, India

**Keywords:** Sublingual temperature, infrared thermometer, tympanic thermometer.

## Abstract

**Background: **Sublingual temperature measurement is a quick and accurate representation of oral temperature and corresponds closely with core temperature. Sub-lingual temperature measurement using non-contact infrared thermometers has not been studied for this purpose and if accurate they would be a reliable and convenient way of recording temperature of a patient very quickly. The aim of the study was to evaluate the utility of recording sublingual temperature using an infrared non-contact thermometer and establish its accuracy by comparing the readings with tympanic thermometer recordings.

**Methods: **This cross-sectional study was carried out in 29 patients (328 paired recordings from sublingual and tympanic sites simultaneously). Subjects were requested to keep their mouth closed for five minutes before recording the temperature. Sublingual recordings were performed for each patient at different times of the day using an infrared thermometer. The infrared thermometer was quickly brought 1cm away from the sublingual part of the tongue and the recordings were then done immediately. Readings were compared with the corresponding tympanic temperature.

**Results: **The non-contact sublingual temperature correlated very closely with tympanic temperature (r=0.86, p<0.001). The mean difference between the infrared sublingual and tympanic temperature was 0.21°C (standard deviation [SD]:0.48°C, 95% confidence interval [CI] of 0.16-0.27). The intra-class correlation co-efficient (ICC) between core and sublingual temperatures was 0.830 (95% CI: 0.794 to 0.861) p<0.001. The sensitivity of sublingual IR (infrared) temperature of 37.65°C was 90% and specificity was 89% for core temperature >38°C.

**Conclusions: **This innovative modification of using the forehead infrared thermometer to measure the sublingual temperature offers an accurate, rapid and non-contact estimation of core temperature.

## Introduction

Body temperature monitoring can be done from multiple sites like oral, rectal, axillary, tympanic, pulmonary, oesophageal, gastrointestinal, bladder and temporal. Sollai *et al* have viewed an ideal thermometer as “An ideal thermometer should: accurately reflect the core body temperature in all age groups; be convenient, easy and comfortable to use; give rapid results; not cause cross infection among patients; not be influenced by room temperature; and be safe and cost- effective”. ^1^ Although oral site is widely utilised for routine temperature recording in adults, rectal site is still considered as a representative of core body temperature. ^2^ Rectal temperature measurement involves an invasive procedure, needs thorough sterilisation after each use and is inconvenient to use. It can be influenced by multiple factors and does not rapidly reflect core body temperature changes. ^2^

The oral site is the most convenient and reliable site for intermittent temperature recordings. ^3^ The oral site is supplied by branches of the external carotid artery. Oral temperature is influenced by many factors like smoking, ^4^ salivation, consumption of cold or warm fluids, chewing gum or mastication, ^4^ and fast breathing, oral disease.

Tympanic site is the most suitable and convenient for non-invasive core body temperature monitoring. The tympanic temperature readings correlate well with the core temperature and is rarely affected with ambient temperature. However, taking tympanic temperature is inconvenient and involves contact of the body with the device which and hence needs cleaning and sterilisation/new probes after each use.

The use of non-contact infrared thermometers (NCIT) has become more prominent over time because of the simplicity and convenience. NCIT for forehead temperature measurement have been very popular but lack accuracy. Non-contact measurements are generally taken over the forehead at 3-15 cm distance. ^5^ Infrared skin thermometers are equivocal in their performance. Hence, they are not routinely used in clinical practice. ^5^

In the search to determine a more practical method of temperature recording, an infrared auditory thermometer was studied to evaluate the temperature measurements at various sites in the oral cavity. ^6^ The sublingual site was found to be the ideal site since the floor of mouth (underside of the tongue) represents core temperature very closely, ^6^ Despite its merits, disposable tips had to be changed for each patient during the study and the study was conducted in healthy individuals, not in patients with fever. ^6^ None of the existing methods mentioned fulfill all the requirements of an ideal thermometer for clinical use.

We innovated the method of measuring sublingual temperature using a forehead infrared thermometer and evaluated its utility in recording the sublingual temperature in a non-contact manner. We established its accuracy by comparing with a tympanic temperature measurement.

## Methods

### Setting

The study was conducted at a tertiary care centre at Kasturba Medical college, Mangalore (Manipal Academy of Higher Education), situated in the south Indian state of Karnataka. The study took place between 2019-2020.

### Participants

Subjects were patients who were enrolled in an ongoing study for the evaluation of 24 hr continuous tympanic temperature in undifferentiated fever. ^7^ Subjects were approached by the primary researcher and informed consent was obtained prior to enrollment into the study. No financial assistance was provided to the participants for enrollment into the study. Preliminary details of participants were obtained from medical records. All participants were subjected to detailed physical examination. Inclusion criteria included age 18-60 years, individuals with an intact tympanic membrane, individuals with normal oral cavity (no evidence of active inflammation/infection). Exclusion criteria were: Individuals who had any ear-related problems, subjects with neurological conditions and multiple clinical co-morbidities, pregnant women and subjects with any infective/inflammatory pathologies of oral cavity.

### Ethical considerations

The study was approved by the Institutional Ethics Committee of Kasturba Medical college, Mangalore. Ethics committee approval number: IEC KMC MLR 05/19/228) and all the subjects provided written informed consent (consent form provided as *Extended data*).

### Procedure

From each patient, 10-12 randomly (with no diurnal preference) timed sublingual temperature recordings were obtained by the primary investigator over a period of 24 hours (waking hours only), using a non-contact thermometer (each recording requiring 5-10 seconds). Corresponding tympanic temperatures (automated minute per minute recording for a duration of 24 hours) were simultaneously recorded and noted with the help of TherCom device. Enrolled subjects were advised not to take food or water for 30 minutes and were requested to keep the mouth closed for five minutes before recording the temperature. The infrared thermometer was kept ready after switching it on and the subjects were asked to quickly open the mouth and lift the tongue exposing the sublingual part of the tongue and hold their breath. The infrared thermometer was quickly brought to 1 cm away from the sublingual part of the tongue and the recordings were then done immediately. The time of the temperature recording with the non contact thermometer was noted. The corresponding temperature recording with tympanic recording was accessed from the recorded data at the exact time, thus eliminating observer bias.

### Thermometers

The sublingual non-contact temperature measurement was done by non-contact infrared digital thermometer (Accu DIGIT F1- BPL (India)). It has an accuracy of ±0.3°C and resolution of 0.1°C at a measuring distance of 1-5 cm. Its measuring range is from 34.9°C-42.2°C. The operating environmental temperature should be between 10°C-40°C. Tympanic temperature was measured by the T-clinic TherCom cartable device (Manufacturer: Innovatec Sensing and Communication, Alicante, Spain. Model No:SN 58021315) to obtain and store real time body temperature data. ^8^ The device has an accuracy of ± 0.2°C with sensor accuracy ± 0.1°C at 37°C and ± 0.2°C at 5°C to 45°C. It has temperature sensors, signal conditioner and amplifier for each channel, an analog to digital converter, a microcontroller, flash memory, and a Bluetooth module. ^8^^,^^9^ The channel was connected by using tympanic thermistor probe, which senses the tympanic temperature and send to the T-clinic TherCom device to store the temperature readings. The complete specification of T-clinicTherCom and the temperature probe were described in previously published literature. ^8^^,^^9^

### Fever definition

There is no universally accepted cut off temperature for defining fever. ^10^ Cut offs of core temperature have varied between 37.5°C to 38°C in various studies. ^10^^,^^11^ The Brighton Collaboration Fever Working Group has defined fever as temperature >38°C. ^12^ We have followed this definition and tympanic temperature of > 38°C was categorized as fever in our study to calculate the sensitivity and specificity of sublingual measurement for the diagnosis fever.

### Statistical analysis

Sample size was calculated for expected sensitivity and specificity of 95% each, 25% prevalence for fever, with a precision and confidence interval of 95% (95% CI). With additional 10% for drop outs/technical issues while recording, the required sample size was calculated to be 325 recordings. Data was analyzed with SPSS version 25. Mean and standard deviation (SD) were calculated for tympanic and sublingual readings. Scatter plots were generated to study the relation between these two variables. The correlation coefficient was calculated using Pearson’s method. An intra-class correlation coefficient was calculated between tympanic and sublingual readings. Mean difference was calculated between these variables. Tympanic temperature of >38°C was defined as fever and receiver operating characteristic (ROC) curve was analyzed for sublingual temperature for predicting fever and positive and negative predictive values, sensitivity and specificity was calculated. Agreement between the two methods for diagnosing fever was assessed by kappa (k) statistics.

## Results

**Figure 1.**
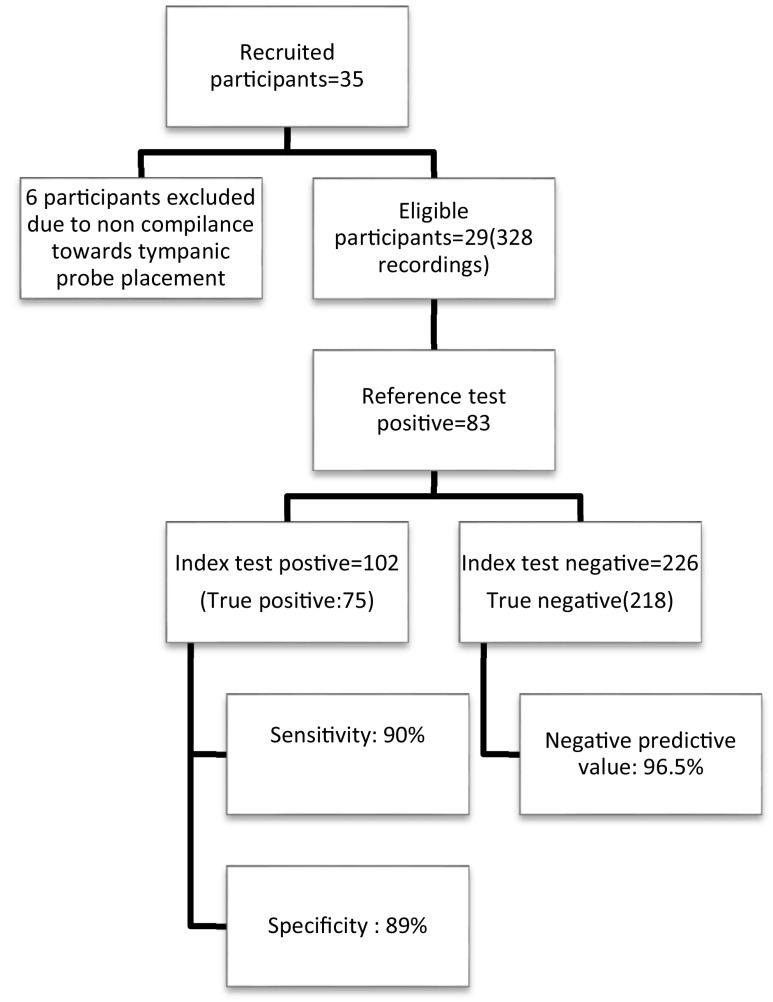
Summary of the study.

**Figure 2.**
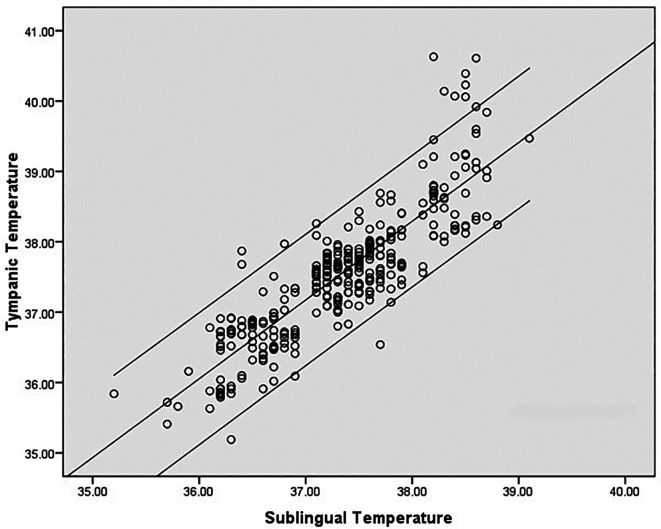
Correlation between tympanic and sublingual temperature (°C) recordings.

In total, 35 participants fulfilling the inclusion criteria were recruited for the study, yet six were excluded due to noncompliance towards tympanic probe placement ( Figure 1). A total of 328 paired recordings from 29 patients were collected. ^13^ The mean age of the subjects was 32.8 ± 11.57 yrs. Medical diagnoses were: dengue fever (22), malaria (4), leptospirosis (1), enteric fever (1), pulmonary tuberculosis (1). There was a high degree of correlation between sublingual infrared (IR) and tympanic temperature; (correlation coefficient r = 0.860, p < 0.001). However, correlation at higher temperatures was poor and sublingual temperature was about 1.5°C less than the tympanic temperature at extreme body temperatures ( Figure 2). Mean difference between infrared sublingual and tympanic temperature was 0.21°C (SD 0.48°C, 95% CI 0.16°C-0.27°C) ( Table 1).

**Table 1.** Basic statistical values of tympanic and sublingual infrared temperature recordings.

Parameters	Tympanic Temperature	Sublingual Infrared Temperature
Mean (°C)	37.54	37.33
SD	0.93	0.71
Minimum (°C)	35.19	35.2
Maximum (°C)	40.36	39.10

SD = standard deviation.

**Figure 3.**
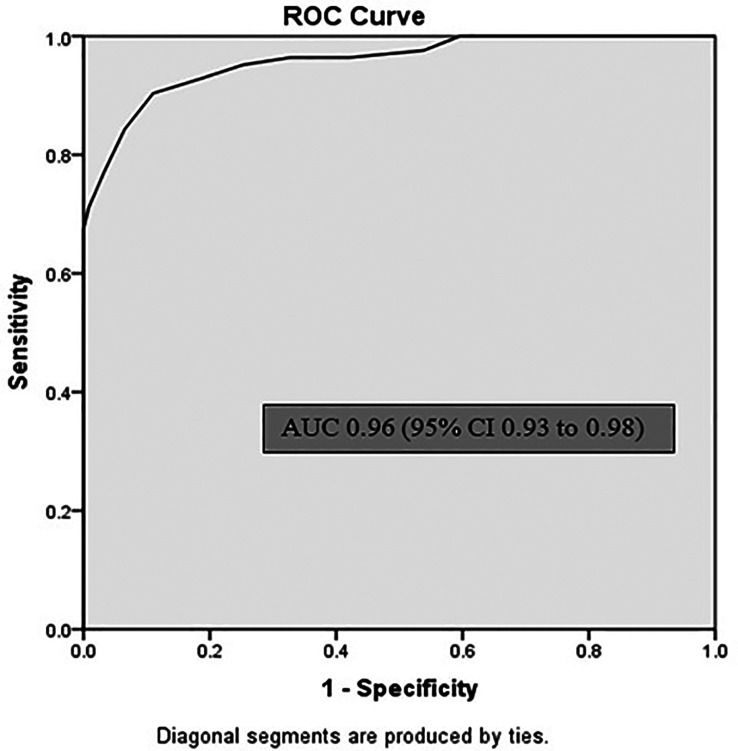
Receiver operating characteristic (ROC) curve for sublingual infrared temperature recordings. AUC = area under curve; CI = confidence interval.

**Figure 4.**
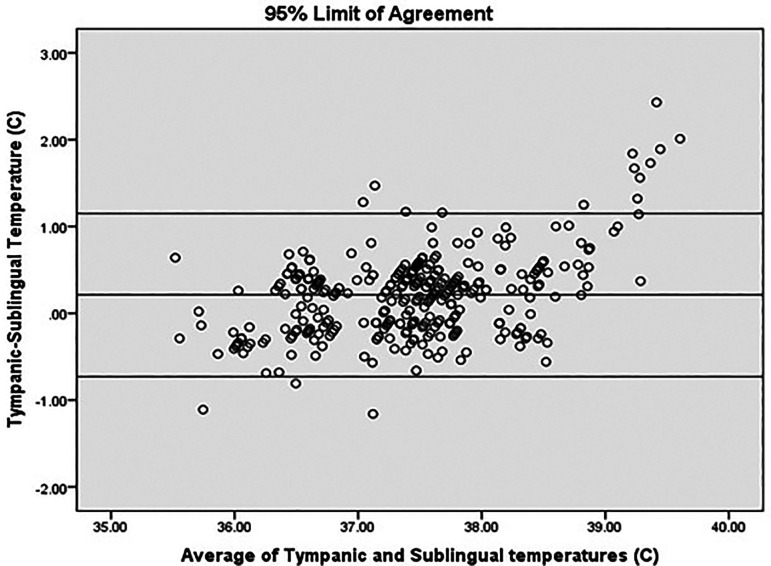
Agreement analysis.

The intra-class correlation co-efficient (ICC) between core and sublingual temperatures was 0.830°C (95% CI: 0.794 to 0.861) p < 0.001. Fever was defined as >38°C. In total, 83 recordings (21.84%) had temperature > 38°C ( Table 2). Sublingual infrared (IR) temperature had area under the curve (AUC) of 0.96 (95% CI 0.93 to 0.98) p < 0.001 for Core temperature of 38°C ( Figure 3). Sensitivity of sublingual IR temperature of 37.65°C was 90%, specificity was 89%, positive predictive value was 73.5% and negative predictive value was 96.5% ( Figure 1). The Kappa coefficient which is a measure of agreement was 0.738, p < 0.001, suggesting a good agreement between sublingual IR and tympanic temperature for diagnosing fever (Temperature > 38°C) ( Figure 4).

**Table 2.** Contingency table for evaluating diagnostic accuracy of sublingual infrared recordings.

		Tympanic recording	
		Fever is present	Fever is absent
**Sublingual IR recording**	Fever is present	75	27
	Fever is absent	8	218

## Discussion

We took a sublingual infrared non-contact thermometer recording by keeping the thermometer about 1 cm from the upturned undersurface of the tongue and compared the reading with tympanic temperature which represents core temperature. Subjects with fever and a variety of underlying clinical condition were included. We observed that there was a statistically significant degree of correlation between the non-contact sublingual infrared recording and the core temperature. There was very good agreement for diagnosing fever (temperature >38°C) between the two methods with sensitivity and specificity of about 90%.

In our study, mean difference was 0.21°C (95% CI 0.16-0.27). 95% limit of agreement (LOA) was −0.73 to 1.15. This value is consistent with other studies reporting temperature differences with various devices/sites. 95% LOA for mercury thermometers for rectal temperature is −0.44°C to 1.27°C and for electronic devices for rectal temperatures −0.46°C to 1.34°C. The inherent biological variations also contribute to the variations. The difference between oral temperature and criterion temperature has been found to be −0.50°C ± 0.31°C in steady state but increased to 1.5°C at temperatures above 39°C. ^14^ Analysis of the LOA plot in our study also revealed that the difference increases above 39°C. Sublingual temperature lags behind the tympanic temperature by an average of 0.21°C. The difference widens at very high body temperatures as shown in the scatter plot ( Figure 2). Similar observations were noted in previous studies also. Oral temperature was compared with rectal temperatures and the oral temperature lagged behind the rectal temperature by about 1.5°C at temperatures of >39°C ^14^ which is similar to our findings.

Sublingual temperatures closely reflect the core temperature, but current methods use contact devices which require sterilisation or change of probe after each use. Using our modification, it is possible to accurately record sublingual temperature quickly without the need to sterilise the equipment. Mass screening can be done quickly and accurately. It may be noted that mass screening with an infrared forehead thermometer is not accurate though convenient. ^9^ This will also result in significant saving of nursing time since the present method of recording takes about 5 minutes, 4-6 times per day for each patient which includes the recording time and the time taken for cleaning the device. In addition, sublingual infrared thermometers are cost effective, easily accessible and can be self-utilised even in a home-based setting.The simpler technique provides an extra advantage that even general population could be easily trained regarding recording methods.

Scientific studies have documented close correlation between sublingual and tympanic temperatures. In one study, sublingual temperature was measured by mercury thermometer and compared with tympanic temperature. ^15^ The mean difference between the two measurements was 0.09 °C (95% CI 0.07-0.12). The clinically acceptable difference for oral temperature compared to reference standard is 0.27°C (0.5° F). ^14^ The difference in our study was 0.21°C, which is well within the clinically acceptable difference.

The non-contact infrared device used in our study is approved for measuring forehead temperature at a distance of 1-5 cm. We used the same device to record the temperature at the sublingual site at a distance of about 1 cm. It is possible to measure from a longer distance if distance to spot ratio is enhanced. If the distance to spot ratio is 12:1, which is possible with many of the routinely used infrared thermometers, readings can be taken from a distance of 1 foot (12 inches) to sample an area of 1 inch diameter of the sublingual area. This will allow the examiner to maintain a safe distance from the patient to avoid droplet infections. A guiding laser beam can be incorporated in the device to accurately select the sublingual area. This will improve the efficacy of mass screening of fever patients which is extremely important in the setting of highly contagious infective diseases. The entire procedure can be incorporated in mobile devices which will allow the mobile devices to work as accurate thermometers.

The main strength of our study is the fact that the measurements were done in varied clinical conditions and in hospital settings. Studies done in healthy volunteers or in controlled laboratory conditions may not be true reflections of their performance in actual clinical scenarios. Moreover, the measurements were correlated with tympanic temperature which is a close measure of core temperature. ^16^

A limitation of our study is the relatively small number of subjects. The study needs to be repeated in a larger number of patients and multiple users to establish its validity. This method may not be accurate in emergency departments and critical care settings since a wide variation of tympanic and oral temperatures have been observed in these settings, probably due to the circulatory compromise. ^17^

Our method satisfies all the requirements of an ideal thermometer defined by Sollali *et al*. ^1^ It has high accuracy for core temperature, it is easy and comfortable to use since it is non-contact, it is rapid since it gives instantaneous readings, does not cause cross infection since it is non-contact and non-invasive, it is not influenced by ambient temperature and is cost effective since initial cost is low and there are no recurring expenses except for the battery.

In conclusion, innovative modification of using the forehead infrared thermometer to measure the sublingual temperature offers an accurate, rapid and non-contact estimation of core temperature.

## Conclusion

The novel modified use of an infrared non-contact thermometer could be better method to record the sublingual temperature of patients quickly and accurately. Implementation of this method could increase the working efficiency of the nursing staff in general patient wards, which is cost effective and there is no need to sterilise the device between patients.

In addition, sublingual temperature recording using an infrared non-contact thermometer could be implemented in mass screening of people in airports, railway stations etc., during global pandemic situations like coronavirus disease 2019 (COVID-19) and Ebola virus disease for example. Because the sublingual temperature closely reflects the core body temperature and could be more accurate and convenient without any influence of ambient temperature. This novel modified method could improve the efficacy and accuracy of body temperature recording.

## Data availability

### Underlying data

Zenodo: A novel method for measuring sublingual temperature using conventional non-contact forehead thermometer. https://doi.org/10.5281/zenodo.5773910. ^13^

This dataset contains the following underlying data:

• Data file 1.xlsx

### Extended data

Zenodo: A novel method for measuring sublingual temperature using conventional non-contact forehead thermometer. https://doi.org/10.5281/zenodo.5773910. ^13^

This dataset contains the following extended data:

• Consent.docx (information sheet and consent form)

Data are available under the terms of the Creative Commons Attribution 4.0 International license (CC-BY 4.0).
