# Supplementary material for: A novel method for measuring sublingual temperature using conventional non-contact forehead thermometer
Source: F1000Res. 2022 Aug 26;11:13. Originally published 2022 Jan 7. [Version 3] doi: 10.12688/f1000research.74876.3 (PMC9396079; doi:10.12688/f1000research.74876.3)
Supplement: Peer review history

# Peer review: Reviewer response for version 2

**DOI:** 10.5256/f1000research.121078.r144075

**Published:** 2022-08-22

**Authors:** Jaya Shanker Tedla

**Affiliations:** 1 Department of Medical Rehabilitation Sciences, College of Applied Medical Sciences, King Khalid University, Abha, Saudi Arabia

The study was a great idea using the forehead non-contact thermometer for measuring the sublingual temperature is innovative in the current times of COVID-19. The authors have a clear idea of what they were doing. The study design was clear, cross-sectional, and suitable for the research question. Study methods were optimal for their findings, and statistical analysis by using the ICC was ideal. 

 I feel in your study; you were establishing concurrent validity (which is defined as " ***Concurrent validity.*** Concurrent validity indicates the amount of agreement between two different assessments. Generally, one assessment is new while the other is well established and has already been proven to be valid.") But it was not mentioned anywhere, and if I am correct, you can include this in the objectives at the end of the introduction. 

 The remaining methodology, results, discussion and conclusion were excellently written by the authors.

Is the work clearly and accurately presented and does it cite the current literature?

Yes

If applicable, is the statistical analysis and its interpretation appropriate?

Yes

Are all the source data underlying the results available to ensure full reproducibility?

Yes

Is the study design appropriate and is the work technically sound?

Yes

Are the conclusions drawn adequately supported by the results?

Yes

Are sufficient details of methods and analysis provided to allow replication by others?

Yes

Reviewer Expertise:

Disability and Rehabilitation

I confirm that I have read this submission and believe that I have an appropriate level of expertise to confirm that it is of an acceptable scientific standard.

# Response

**Published:** 2022-08-24

**Authors:** ARJUN R GUPTA

**Affiliations:** Manipal University, India

Thank you for your valuable suggestion sir.

 We have tried to implement your suggestion regarding our article.

 We have made following suitable changes in the article.

**Reviewer Comments; followed by the response (in bold) by Authors:**

 I feel in your study; you were establishing concurrent validity (which is defined as " Concurrent validity. Concurrent validity indicates the amount of agreement between two different assessments. Generally, one assessment is new while the other is well established and has already been proven to be valid.") But it was not mentioned anywhere, and if I am correct, you can include this in the objectives at the end of the introduction. 

**We established concurrent validity of sublingual temperature using a non-contact thermometer by comparing it with tympanic temperature measurement which is an established and standard method of recording the core temperature.**

# Peer review: Reviewer response for version 2

**DOI:** 10.5256/f1000research.121078.r124311

**Published:** 2022-02-28

**Authors:** Balakrishnan Valliyot

**Affiliations:** 1 Department of General Medicine, Academy of Medical Sciences, Kannur, India

Sir,

 Thank you for sending the revised articles. I have carefully reviewed the modification and corrections made by the authors and the authors made necessary modifications and revised the article thoroughly to the satisfaction of the reviewer. This modified article can be accepted for publication.

 Prof. Dr. Balakrishnan Valliyot

Is the work clearly and accurately presented and does it cite the current literature?

Yes

If applicable, is the statistical analysis and its interpretation appropriate?

Yes

Are all the source data underlying the results available to ensure full reproducibility?

Yes

Is the study design appropriate and is the work technically sound?

Yes

Are the conclusions drawn adequately supported by the results?

Yes

Are sufficient details of methods and analysis provided to allow replication by others?

Yes

Reviewer Expertise:

Internal medicine, Non-communicable diseases, Clinical medicine, Type 2 diabetes

I confirm that I have read this submission and believe that I have an appropriate level of expertise to confirm that it is of an acceptable scientific standard.

# Peer review: Reviewer response for version 1

**DOI:** 10.5256/f1000research.78677.r118955

**Published:** 2022-01-11

**Authors:** Balakrishnan Valliyot

**Affiliations:** 1 Department of General Medicine, Academy of Medical Sciences, Kannur, India

The research topic is innovative since human body temperature is conventionally measured using a mercury thermometer placed under the sublingual area. This method has several demerits and using a non-contact digital thermometer will overcome some of these demerits. This will offer more accuracy, more hygienic practices, and avoidance of mercury. Hence, this method has future potential in clinical medicine. But the following points need more clarifications:

 1. The study mentioned that monitoring time is 24 hours, but how much time is required for a single recording needs more clarification. 

 2. Whether this can be recorded by patients or needs more technical support from staff and whether it will be cost-effective if a more sophisticated device is needed for interpretation (note ref 12, 13).

 3. The introduction part needs thorough modifications and language editing since certain paragraphs contain statistical comparisons and nearly ten references of other studies were quoted in the introduction. The introduction section needs rewriting.

Is the work clearly and accurately presented and does it cite the current literature?

Yes

If applicable, is the statistical analysis and its interpretation appropriate?

Yes

Are all the source data underlying the results available to ensure full reproducibility?

Yes

Is the study design appropriate and is the work technically sound?

Yes

Are the conclusions drawn adequately supported by the results?

Yes

Are sufficient details of methods and analysis provided to allow replication by others?

Yes

Reviewer Expertise:

Internal medicine, Non-communicable diseases, Clinical medicine, Type 2 diabetes

I confirm that I have read this submission and believe that I have an appropriate level of expertise to confirm that it is of an acceptable scientific standard, however I have significant reservations, as outlined above.

# Response

**Published:** 2022-02-16

**Authors:** ARJUN R GUPTA

**Affiliations:** Manipal University, India

Thank you for your valuable suggestions sir. We are humbled by your positive views on our article. We have also taken your queries into consideration sir. We have tried to address all of your concerns regarding our article.

 We have made following suitable changes. Reviewer Comments; followed by the response (in bold) by Authors:

- The study mentioned that monitoring time is 24 hours, but how much time is required for a single recording needs more clarification.

**Each sublingual recording required about 5-10 seconds to complete. Corresponding core temperature recording was an automated process via Thercom device (automatic updating of tympanic temperature happens every minute for 24 hours, and was visible in the device screen).**

  

- Whether this can be recorded by patients or needs more technical support from staff and whether it will be cost-effective if a more sophisticated device is needed for interpretation (note ref 12, 13).

**The recording can be easily done by patient or any other personnel, given he/she is been provided with basic instructions before recording.**

  

- The introduction part needs thorough modifications and language editing since certain paragraphs contain statistical comparisons and nearly ten references of other studies were quoted in the introduction. The introduction section needs rewriting.

**The introduction part has been modified as suggested.**

 We intend to hear back from you soon sir. Kindly provide your valuable suggestions if any, for further betterment of our article.
